# Nitrogen limitation-induced adaptive response and lipogenesis in the Antarctic yeast *Rhodotorula mucilaginosa* M94C9

**DOI:** 10.3389/fmicb.2024.1416155

**Published:** 2024-08-05

**Authors:** Miguel Rosas-Paz, Alberto Zamora-Bello, Nayeli Torres-Ramírez, Diana Villarreal-Huerta, Lucero Romero-Aguilar, Juan Pablo Pardo, Mohammed El Hafidi, Georgina Sandoval, Claudia Segal-Kischinevzky, James González

**Affiliations:** ^1^Departamento de Biología Celular, Facultad de Ciencias, Universidad Nacional Autónoma de México, Mexico City, Mexico; ^2^Posgrado en Ciencias Biológicas, Unidad de Posgrado, Circuito de Posgrados, Ciudad Universitaria, Mexico City, Mexico; ^3^Posgrado en Ciencias Bioquímicas, Unidad de Posgrado, Ciudad Universitaria, Mexico City, Mexico; ^4^Departamento de Bioquímica, Facultad de Medicina, Universidad Nacional Autónoma de México, Mexico City, Mexico; ^5^Departamento de Biomedicina Cardiovascular, Instituto Nacional de Cardiología Ignacio Chávez, Mexico City, Mexico; ^6^Laboratorio de Innovación en Bioenergéticos y Bioprocesos Avanzados, Unidad de Biotecnología Industrial, Centro de Investigación y Asistencia en Tecnología y Diseño del Estado de Jalisco A. C., Guadalajara, Mexico

**Keywords:** *Rhodotorula mucilaginosa*, lipogenesis, nitrogen starvation, oleaginous yeast, neutral lipid production, adaptive stress response

## Abstract

The extremotolerant red yeast *Rhodotorula mucilaginosa* displays resilience to diverse environmental stressors, including cold, osmolarity, salinity, and oligotrophic conditions. Particularly, this yeast exhibits a remarkable ability to accumulate lipids and carotenoids in response to stress conditions. However, research into lipid biosynthesis has been hampered by limited genetic tools and a scarcity of studies on adaptive responses to nutrient stressors stimulating lipogenesis. This study investigated the impact of nitrogen stress on the adaptive response in Antarctic yeast *R. mucilaginosa* M94C9. Varied nitrogen availability reveals a nitrogen-dependent modulation of biomass and lipid droplet production, accompanied by significant ultrastructural changes to withstand nitrogen starvation. *In silico* analysis identifies open reading frames of genes encoding key lipogenesis enzymes, including acetyl-CoA carboxylase (Acc1), fatty acid synthases 1 and 2 (Fas1/Fas2), and acyl-CoA diacylglycerol O-acyltransferase 1 (Dga1). Further investigation into the expression profiles of *RmACC1*, *RmFAS1*, *RmFAS2*, and *RmDGA1* genes under nitrogen stress revealed that the prolonged up-regulation of the *RmDGA1* gene is a molecular indicator of lipogenesis. Subsequent fatty acid profiling unveiled an accumulation of oleic and palmitic acids under nitrogen limitation during the stationary phase. This investigation enhances our understanding of nitrogen stress adaptation and lipid biosynthesis, offering valuable insights into *R. mucilaginosa* M94C9 for potential industrial applications in the future.

## 1 Introduction

Non-*Saccharomyces* yeasts represent a growing area with significant potential for the bioeconomy, emerging as producers of value-added compounds (Segal-Kischinevzky et al., 2022). Oleaginous yeasts (OY) are emerging as promising candidates for the production of biofuels and oleochemicals due to their ability to accumulate cellular lipid content exceeding 20% of dry cell mass (Sitepu et al., 2014). However, the current identification of OY is less than 9% of the total known yeast species, without considering OY strains of the same species (Abeln and Chuck, 2021; Salvador López et al., 2022; Poontawee et al., 2023). Furthermore, the ability of OY to accumulate lipids and produce specific fatty acid profiles depends on the strain, environmental conditions, and adaptations in metabolic pathways, underscoring the presence of unidentified mechanisms and factors that govern lipogenesis.

**Figure 1:**
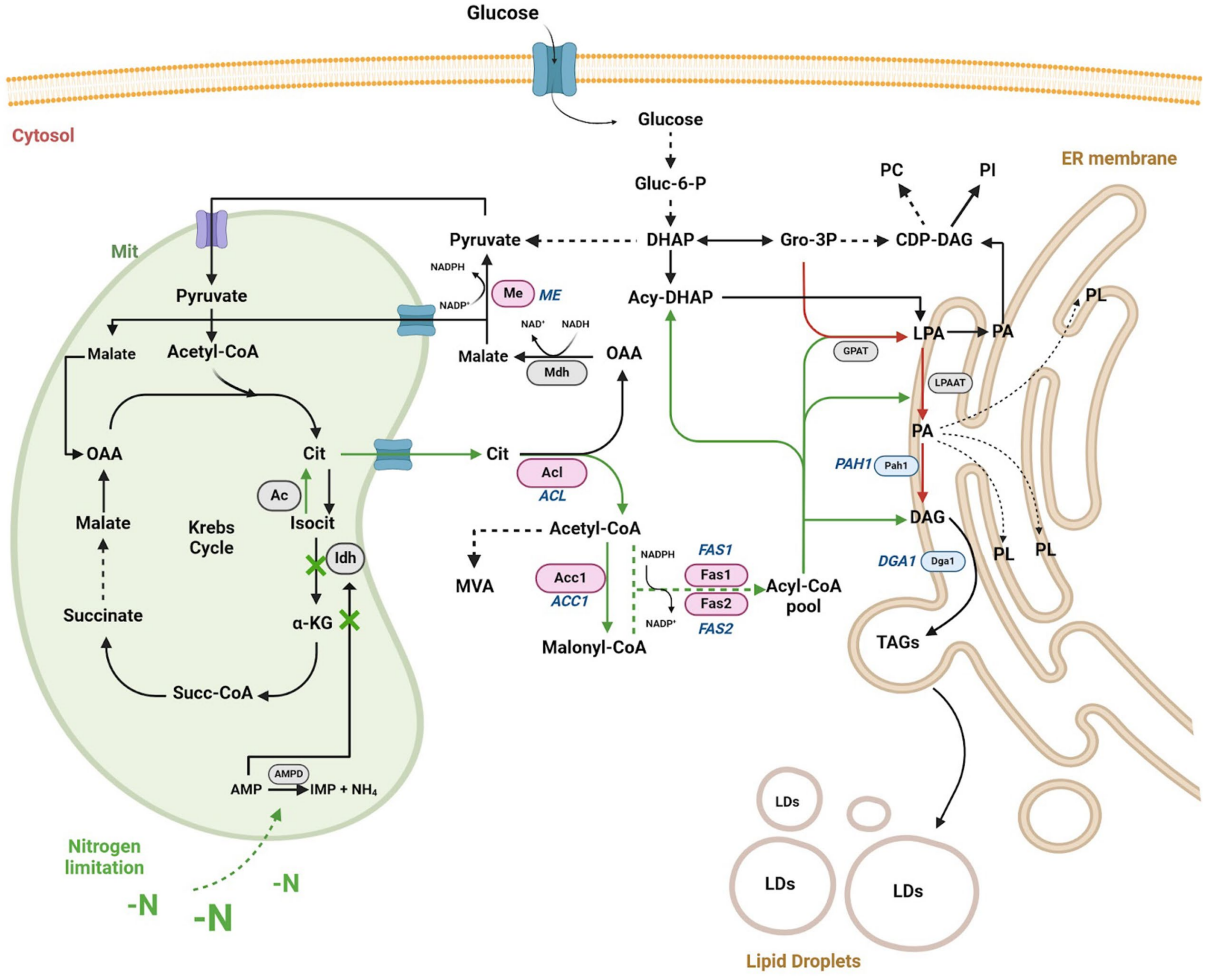
Schematic representation of the lipid biosynthesis pathway in OY under nitrogen limitation or starvation conditions. Under nitrogen stress conditions, TAGs (triacylglycerols) production increases via Gro-3P/LPA/PA/DAG (depicted by red arrows). During nitrogen limitation or starvation (-N) conditions, mitochondrial citrate (Cit) translocates to the cytosol via the cit/malate shuttle, augmenting the pool of acetyl-CoA and NADPH in the cytosol, thereby enhancing TAG biosynthesis (depicted by green arrows). The key enzymes initiating TAG biosynthesis under -N conditions are represented by pink ovals (Acl, Acc1, Me, and Fas1/Fas2), with gene names in italics (ACL, ACC1, ME, and FAS1/FAS2). Enzymes primarily involved in DAG (diacylglycerol) and TAG production in the endoplasmic reticulum (ER) are illustrated by blue ovals (Pah1 and Dga1), with gene names in italics (PAH1 and DGA1). Other relevant enzymes in the pathway are denoted by gray ovals (AMPD, Idh, Ac, Mdh, GPAT, LPAAT). Accumulation of TAGs by both pathways contributes to the formation of lipid droplets (LDs) within the cell. Metabolites are depicted in black letters. Dashed arrows signify specific catalytic steps. Abbreviations include oxaloacetic acid (OAA), adenosine monophosphate (AMP), inosine monophosphate (IMP), ammonium ion (NH_4_), isocitrate (Isocit), mevalonate (MVA), dihydroxyacetone phosphate (DHAP), acyl-dihydroxyacetone phosphate (Acyl-DHAP), glycerol-3-phosphate (Gro-3P), lysophosphatidic acid (LPA), phosphatidic acid (PA), glucose-6-phosphate (Gluc-6-P), α-ketoglutarate (α-KG), succinyl CoA (succ-CoA), CDP-diacylglycerol (CDP-DAG), phosphatidylcholine (PC), phospholipids (PL), and phosphatidylinositol (PI), AMP-deaminase (AMPD). Based on Caporusso et al., 2021; Segal-Kischinevzky et al., 2022. Created using BioRender.com, accessed on 26 March 2024.

The evidence supports the notion that OY respond to nutritional stress factors, such as nitrogen limitation, by accumulating high levels of triacylglycerols (TAGs) as long as a carbon source is available to support lipogenesis (Ageitos et al., 2011; Papanikolaou and Aggelis, 2011; Taccari et al., 2012). Under these conditions (Figure 1), adenosine monophosphate (AMP) in mitochondria is converted to inosine monophosphate (IMP) and ammonium ions (NH_4_^+^) through the AMP-deaminase (AMPD) activity (Dourou et al., 2018). The resulting NH_4_^+^ provides an additional nitrogen source crucial for cellular component synthesis following extracellular nitrogen limitation (Ratledge and Wynn, 2002). The decrease in mitochondrial AMP concentration negatively affects the isocitrate dehydrogenase (Idh) activity, accumulating mitochondrial citrate. Lipogenesis is initiated when mitochondrial citrate is transported to the cytosol, exchanging citrate for malate. Carbon flux is directed predominantly toward triacylglycerol biosynthesis through the initial enzymes, including ATP citrate lyase (Acly), acetyl-CoA carboxylase (Acc1), and fatty acid synthases 1 and 2 (Fas1/Fas2), resulting in the subsequent storage of TAGs within the cell’s lipid droplets (LDs) that previously involves the acylation of diacylglycerols by acyl-CoA diacylglycerol O-acyltransferase 1 (Dga1) (Tehlivets et al., 2007; Tang et al., 2015; Pomraning et al., 2016). Despite these findings, the utilization of OY-derived lipids for industrial biofuel synthesis remains challenging due to the associated high costs of microbial biomass production (Koutinas et al., 2014).

Antarctica, with its extreme environmental conditions and nutrient scarcity, represents a source of OY that exhibits adaptive capabilities (Segal-Kischinevzky et al., 2022). Viñarta et al. (2016) observed Antarctic OY, including *Rhodotorula glutinis*, *R. glacialis*, and *R. laryngis*, accumulating lipid yields surpassing 70% of dry cell mass under nitrogen limitation conditions (Viñarta et al., 2016). Villarreal et al. (2018) reported Antarctic yeasts with a high linoleic acid content, suggesting their suitability for industrial-scale fatty acid production (Villarreal et al., 2018). Therefore, the Antarctic OY of the genus *Rhodotorula* are considered to have biotechnological potential, but further studies are required focused on elucidating the mechanisms of nitrogen stress adaptation and lipid accumulation (González et al., 2020), bringing with them new knowledge and development for industrial applications, such as commercially valuable fatty acids (omega-3, omega-6, linoleic acid and ricinoleic acid) or TAGs for biodiesel production.

In this investigation, we characterize *R. mucilaginosa* M94C9 isolated from the soil of Snow Island in Antarctica (Troncoso et al., 2017), with a primary emphasis on growth and biomass production in response to nitrogen availability. By elucidating the proportion of carbon/nitrogen required for sustained growth and examining the temporal dynamics of LDs accumulation, we investigate the impact of nitrogen availability on lipogenesis. Additionally, we identify and analyze the expression profile of genes encoding Acc1, Fas1, Fas2, and Dga1, pivotal enzymes in lipid biosynthesis. Furthermore, we assess the biotechnological potential of this red yeast as feedstock for oleochemical production by analyzing fatty acid profiles and evaluating the conversion rate of TAGs to free fatty acid ethyl esters. This study contributes to the understanding of strategies for lipid production and provides insights into yeast adaptation responses to nitrogen stress.

## 2 Materials and methods

### 2.1 Strain and culture media

The *R. mucilaginosa* M94C9 strain was isolated from soil samples from Snow Island, part of the South Shetland Archipelago, Antarctica, by the research group of Dr. Marcelo Baeza from the Universidad de Chile (Troncoso et al., 2017).

Cells were grown and preserved in Yeast Peptone Dextrose agar (YPD-agar) (1% yeast extract, 2% peptone, 2% glucose, and 2% agar). Cells were cultured in 50 mL (liquid volume 1:5) of YPD medium at 180 rpm and 28°C (overnight), representing seed condition (time 0 h). After cultivation, the cells were recovered by centrifugation for 5 min at 1,100 × g at 4°C, washed twice, and suspended in sterile distilled water. These cells were used as an inoculum for YPD and four nitrogen-limited glucose-based media with different carbon/nitrogen (C/N) ratios, varying the nitrogen source concentration. All media compositions (C/N) were implemented with glucose excess (0.55 M) and supplemented with Yeast Nitrogen Base without nitrogen source (YNB). Ammonium sulfate [(NH₄)₂SO₄] was used as the nitrogen source, and the C/N ratios were calculated as follows:

$C/N=\frac{\frac{m_{C}\cdot n_{C}\cdot AWC}{MW_{C}}}{\frac{m_{N}\cdot n_{N}\cdot AWN}{MW_{N}}}$

where:

*m*_C_: mass of the carbon source in grams.

*n*_C_: number of carbon atoms in the carbon source (glucose).

AWC: atomic weight of carbon (12.01 g/mol).

MW_C_: molecular weight of the carbon source.

*m*_N_: mass of the nitrogen source in grams.

*n*_N_: number of nitrogen atoms in the nitrogen source (ammonium).

AWN: atomic weight of nitrogen (14.01 g/mol).

MW_N_: molecular weight of the nitrogen source.

Thus, the ammonium sulfate concentrations were varied as follows: 5 g/L (C/N_40:1_), 0.16 g/L (C/N_1200:1_), 0.08 g/L (C/N_2400:1_), and without nitrogen (-N). The provided equation explicitly indicates that both the number of C and N atoms from different sources, along with their respective atomic weights, must be considered when calculating the C/N ratio if it is to be understood as an atomic ratio.

### 2.2 Growth curves and biomass

Cells were inoculated at 0.05 Optical Density (OD) at 600 nm in the growth media described above (YPD, C/N_40:1_, C/N_1200:1_, C/N_2400:1_, and -N). Cultures in flasks (liquid volume 1:5) were incubated at 28°C with continuous shaking at 180 rpm, and cell growth was monitored by measuring OD every two hours. The exponential phase was considered at 12 h of culture, while the early stationary and stationary phases were defined at 18 h and 48 h, respectively. No growth phases are defined for cells incubated in the -N condition. Aliquots were collected in growth intervals of 0, 24, 48, and 72 h for biomass determination. Cells were centrifuged at 1,100 × g, washed twice, and suspended 1:1 (w/v) in distilled water. Samples were placed on a pre-weighed aluminum container and dried at 70°C during 72 h. The biomass corresponds to the dry weight of the cells, which is presented as the average of three independent experiments.

### 2.3 Transmission electron microscopy

Cells were fixed overnight at 4°C with 4% glutaraldehyde in 0.2 M PIPES buffer (pH 6.8) containing 0.2 M sorbitol, 2 mM MgCl_2_, and 2 mM CaCl_2_. Next, samples were rinsed with water and post-fixed in 2% potassium permanganate for 45 min at room temperature. Cells were contrasted with 1% uranyl acetate and dehydrated in graded ethanol series from 30 to 100%. After dehydration, samples were infiltrated and embedded with EPON. Ultrathin sections of 60 nm were cut using the ultramicrotome Leica Ultracut UCT, prepared on copper grids covered with formvar, and contrasted with uranyl acetate and lead citrate. Sections were observed under a Jeol 1,010 electron microscope at 80 kV. Digital images were captured with a Hamamatsu camera (Hamamatsu Photonics K. K., Japan).

### 2.4 Confocal microscopy

According to Aguilar et al. (2017), cells were collected and adjusted at 5 OD in distilled water. Next, cells were incubated in 2 μM of the fluorescent dye 4,4-difluoro-4-bora-3a,4a-diaza-s-indacene (Bodipy 493/503^Ⓡ^) during 10 min. Cells were washed twice with sterile distilled water, and prepared in Silane-Prep slides with Calcofluor-White (100 ng/mL). Then, samples were coverslipped and photographed with a confocal microscope (Zeiss LSM5 Pascal, Carl Zeiss GmbH, Göttingen, Germany), using an oil immersion 100 × N.A. 1.3 objective. Bodipy and Calcofluor-White fluorescence intensity were detected using excitation and emission wavelengths of 493–503 nm and 300–412 nm, respectively. Images were analyzed using ImageJ software (Rasband, W.S., ImageJ, U. S. National Institutes of Health, Bethesda, Maryland, United States).

### 2.5 Lipid index

The total neutral lipid content of cells was determined following a liquid fluorescence recovery protocol described by Romero-Aguilar et al. (2018), using Bodipy 493/503^Ⓡ^. Aliquots of 1 mL of cell culture were resuspended and fixed with 3.7% formaldehyde for 15 min using a vortex mixer. Cells were washed twice by centrifuging them at 16,000 × g for 1 min at room temperature, discarding the supernatant, and suspending the pellet in distilled water. Cells were adjusted to an OD of 5 with distilled water and stored at 4°C.

The fluorescence recovery assay was performed with a spectrofluorometer programmed for an excitation wavelength of 485 nm and emission at 510 nm (fluorescence), and OD at 600 nm (absorbance). In a 96-well black-clear bottom plate, 200 μL of Bodipy (5 μM) and 0.5 M potassium iodide (external fluorescence quencher) solution was added to each well. The fluorescence and OD of successive additions of 5 μL/well of fixed resuspended cells were measured by incubating the cells for 5 min at room temperature (25°C) with continuous and gentle agitation each time. In total, 4 additions of 5 μL each were made. For each sample in the microplate, the slopes for the fluorescence and absorbance against the volume of each successive addition were obtained, and the “lipid index” was calculated as the quotient between the fluorescence and OD slopes.

### 2.6 Identification of selected lipogenesis-related genes

The prediction of genes in the *R. mucilaginosa* genome needs to be better annotated. Therefore, the orthologous of selected lipogenesis-related genes were compared to identify the genes in *R. mucilaginosa*. The sequences of *ACC1*, *FAS1*, *FAS2*, and *DGA1* orthologous genes previously identified in *Saccharomyces cerevisiae*, *Yarrowia lipolytica*, and *Rhodotorula toruloides* were recovered (Table 1). The systematic names of these genes were obtained in the GenBank database.

**Table 1:** Systematic names for retrieving orthologous gene sequences of Acc1, Fas1, Fas2, and Dga1 in the GenBank.

		Systematic names	
Protein names	*S. cerevisiae*	*Y. lipolytica*	*R. toruloides*
Acetyl-CoA carboxylase 1 (Acc1)	YNR016C	YALI0C11407g	RHTO_02004
Fatty acid synthase subunit 1 (Fas1)	YKL182W	YALI0B15059g	RHTO_02032
Fatty acid synthase subunit 2 (Fas2)	YPL231W	YALI0B19382g	RHTO_02139
Acyl-CoA diacylglycerol O-acyltransferase 1 (Dga1)	YOR245C	YALI0E32769g	RHTO_01962

In addition, the amino acid sequences of the previously reported proteins Acc1, Fas1, Fas2, and Dga1 of *R. toruloides* (Zhu et al., 2012) were recovered and used for BLAST analysis in MycoCosm.^1^ The gene sequences were identified in the *R. mucilaginosa* ATCC58901 v1.0 genome. Coding sequences were translated to obtain amino acid sequences, which were then used to perform multiple alignments using Clustal Omega.^2^ The genes identified were named *RmACC1* (Transcript Id: 689533/Protein Id: 689423), *RmFAS1* (Transcript Id: 737309/Protein Id: 737199), *RmFAS2* (Transcript Id: 693675/Protein Id: 693565), and *RmDGA1* (Transcript Id: 688844/Protein Id: 688734).

### 2.7 RNA extraction

Total RNA was extracted as previously described by Schmitt et al., 1990. Cultures were grown overnight in rich media YPD (seed culture or time 0 h) and then inoculated in C/N_40:1_ or -N media (lipogenic conditions) for 18 or 48 h (OD ∼ 1). Briefly, cells were washed twice, centrifuged at 1,100 × g, and mechanically disrupted using a vortex mixer with sterile glass microbeads (425–600 μm) previously incubated in phenol pH 4.5. Then, the samples were incubated for 5 min at 65°C and vortexed for 30 s twice. The mixture was chilled and centrifuged to separate the aqueous and phenol phases. The aqueous phase was extracted with phenol:chloroform:isoamyl alcohol (25:24:1) and chloroform:isoamyl alcohol twice. RNA precipitation was performed by adding sodium acetate and ethanol and incubating at −20°C for 30 min, followed by centrifugation (16,000 × g). The resulting pellet was washed, dried, and resuspended in RNase-free water. RNA integrity was verified by electrophoresis in a 1% denaturing agarose gel.

### 2.8 Analysis of gene expression

Total RNA was incubated with DNaseI (RQ1 RNase-Free DNase, Promega) to remove contaminating genomic DNA. cDNA synthesis reactions were performed using the RevertAid H Minus First Strand cDNA Synthesis kit (Thermo Scientific, Waltham, MA, United States) following the manufacturer’s recommendations and using oligo (dt) primer. Quantitative real-time PCR (RT-qPCR) was performed using the standard curve method with specific deoxyoligonucleotides for the genes encoding putative Acetyl-CoA carboxylase 1 (*RmACC1*, Id: 689533), Fatty acid synthase subunit 1 (*RmFAS1*, Id: 737309), Fatty acid synthase subunit 2 (*RmFAS2*, Id: 693675), and Acyl-CoA diacylglycerol O-acyltransferase 1 (*RmDGA1*, Id: 688844). Deoxyoligonucleotides were initially screened for the absence of dimers formation and cross-hybridization. Deoxyoligonucleotide pairs with 100% amplification efficiencies were used (Table 2). RT-qPCR analysis was performed using a Rotor-Gene Q (Qiagen) machine. The detection dye was SYBR Green using KAPA SYBR Fast kit (Roche) following the profile settings: 94°C for 5 min (1 cycle), 94°C for 15 s, 58°C for 20 s, and 72°C for 20 s (35 cycles). Transcripts were normalized relative to the *RmACT1* transcript (Id: KY937967) quantities with the standard curve method. Data was represented as a relative expression to *RmACT1* and transformed into log_2_ values (fold change) for visualization in a heatmap. The data shown are the mean values ± SD of four biological replicates and two technical replicates. Statistical analysis was performed using a two-way ANOVA, which was carried out on GraphPad Prism 10.2.3 (GraphPad Software Inc.).

**Table 2:** Deoxyoligonucleotides used for qPCR analysis.

Target	Primer name	Sequence 5′–3′	Amplicon size (bp)
*RmACT1*	*RmACT1Fw*	AAGGTCAACCGCGAGAAGAT	72
	*RmACT1Rv*	AGCGTACAGCGAGAGGAC	
*RmACC1*	*RmACC1Fw*	GGCATGTCGAACGAGCTGAA	126
	*RmACC1Rv*	TCTTCGAGGACGATGCGGAT	
*RmFAS1*	*RmFAS1Fw*	TATCACCGACCCGTATGAGC	108
	*RmFAS1Rv*	GTCGCGGAACATCTTCGAGA	
*RmFAS2*	*RmFAS2Fw*	TTCTCCGCCACCTTCTTGCA	104
	*RmFAS2Rv*	GTCTTGGCGAGGAAGTAGGA	
*RmDGA1*	*RmDGA1Fw*	TCTCATCGTCGCCTACCTCA	123
	*RmDGA1Rv*	ACTGGGTAGTACCCGGCAAA	

### 2.9 Fatty acid profiles

Cells were cultured, washed twice, and collected from C/N_40:1_ at and 48 h of growth. Total lipids were extracted from yeast homogenate in the presence of 50 μg of heptadecanoic acid as an internal standard, using chloroform-methanol (2:1, v/v) containing 0.002% of butylated hydroxytoluene (BHT) (Folch et al., 1957). The obtained lipid residue was transesterified to their fatty acid methyl esters (FAMEs) by heating at 80°C for 2 h with MeOH, containing 2% concentrated H_2_SO_4_ and 0.005% BHT. FAMEs were separated and identified by gas–liquid chromatography in a Shimadzu chromatography, fitted with a 25 m × 0.25 mm i.d. fused silica capillary column, which was coated with DB-FATWAX (film thickness 0.25 μm). The concentration and composition of FAMEs were evaluated by gas–liquid chromatography, as described previously (el Hafidi et al., 2001). Each fatty acid was identified by comparison its retention time with that of its corresponding standard. The quantification of fatty acids was performed by calculating the ratio of the peak area of the identified fatty acid to the peak are of the internal standard, then multiplying by 50 µg and dividing the amount of each fatty acid by 10 mg of total protein, expressing the results as a ratio of mg of lipid per mg of protein. Total protein concentration was determined by the Bradford assay using bovine serum albumin as standard (Bradford, 1976). All experiments were repeated three times, and the results were expressed as mean ± SEM.

### 2.10 Lipid extraction and thin layer chromatography analysis

The lipid content was measured by extracting lipids from the cells using the method proposed by Vargas-Sánchez, 2019. For this purpose, cells were cultivated in a lipogenic condition, as previously described Niehus et al. (2018). Cells were collected, washed twice, and concentrated on pre-weighed 250 mL centrifuge bottles. Then, 100 mL of 4 M HCl was added and vigorously shaken using a vortex. Subsequently, the bottles were placed in an orbital shaker at 60°C and 220 rpm for 6 h. Later, a chloroform:methanol mixture (2:1 v/v) was added, and the samples were shaken for two hours. After that, the bottles were centrifuged to separate the phases, and the upper phase corresponding to the aqueous phase and biomass was discarded. Finally, lipid extracts were recovered by evaporation of the bottom layer solvent. The content of the bottles corresponds to the total lipid content. Lipid percentage was calculated using biomass and lipid content.

Thin Layer Chromatography (TLC) was performed to determine the type of lipids in the obtained oil. For the TLC analysis, 5 mg of the yeast cell oil was diluted in 100 μL of ACS-grade hexane. Subsequently, 0.15, 0.10, and 0.05 mg of the diluted oil were spotted onto a 10 × 10 cm TLC plate (Silica gel on TLC AL foils, SIGMA). A mixture of standards containing monoglycerides (MAGs), diglycerides (DAGs), triglycerides (TAGs), oleic acid, and methyl oleate was also spotted on the plate as markers. Control samples of 0.15 mg and 0.10 mg of hexane-diluted commercial oil were also analyzed. The migration was conducted using a mobile phase of hexane:ethyl ether:acetic acid mixture (85:15:1 v/v). TAGs were visualized using an iodine reagent after the samples migrated on the TLC plate.

### 2.11 Fatty acid ethyl esters production by enzymatic transesterification

According to Rivera et al. (2009), the immobilized lipase 435 from Novozymes was used as a catalyst for free Fatty Acid Ethyl Esters (FAEE or biodiesel) production through transesterification. For this purpose, the yeast oil sample was dissolved in ethanol, mixed with an immobilized lipase (Novozymes 435) in a glass vial. The reaction was carried out at 300 rpm with magnetic agitation during 48 h at 45°C. To analyze whether the conversion of TAGs to FAEEs was adequate, a TLC was performed using yeast oil samples treated with the lipase for 48 h and compared with untreated yeast oil samples (Rivera et al., 2009).

## 3 Results

### 3.1 Effect of nitrogen limitation on the growth and biomass of *R. mucilaginosa* M94C9

Nitrogen is a crucial nutrient for synthesizing various biomolecules, including nucleotides, amino acids, polyamines, glutathione, and other biologically important compounds (Kurmi and Haigis, 2020). Nitrogen starvation causes stress, maintaining a quiescent state associated with the stationary phase (An et al., 2014). Under these conditions, some yeasts, such as *S. cerevisiae*, continue to consume glucose, downregulating fermentation enzymes (Li et al., 2015). However, the biosynthesis of neutral lipids predominates in OY when glucose is abundant under nitrogen-limited conditions (Papanikolaou and Aggelis, 2011; Shi et al., 2017). Then nitrogen starvation will eventually arrest yeast in the G1 phase of the cell cycle. Therefore, the growth and biomass production of *R. mucilaginosa* M94C9 were characterized under different nitrogen concentrations to find a lipogenic condition with significant biomass production.

**Figure 2:**
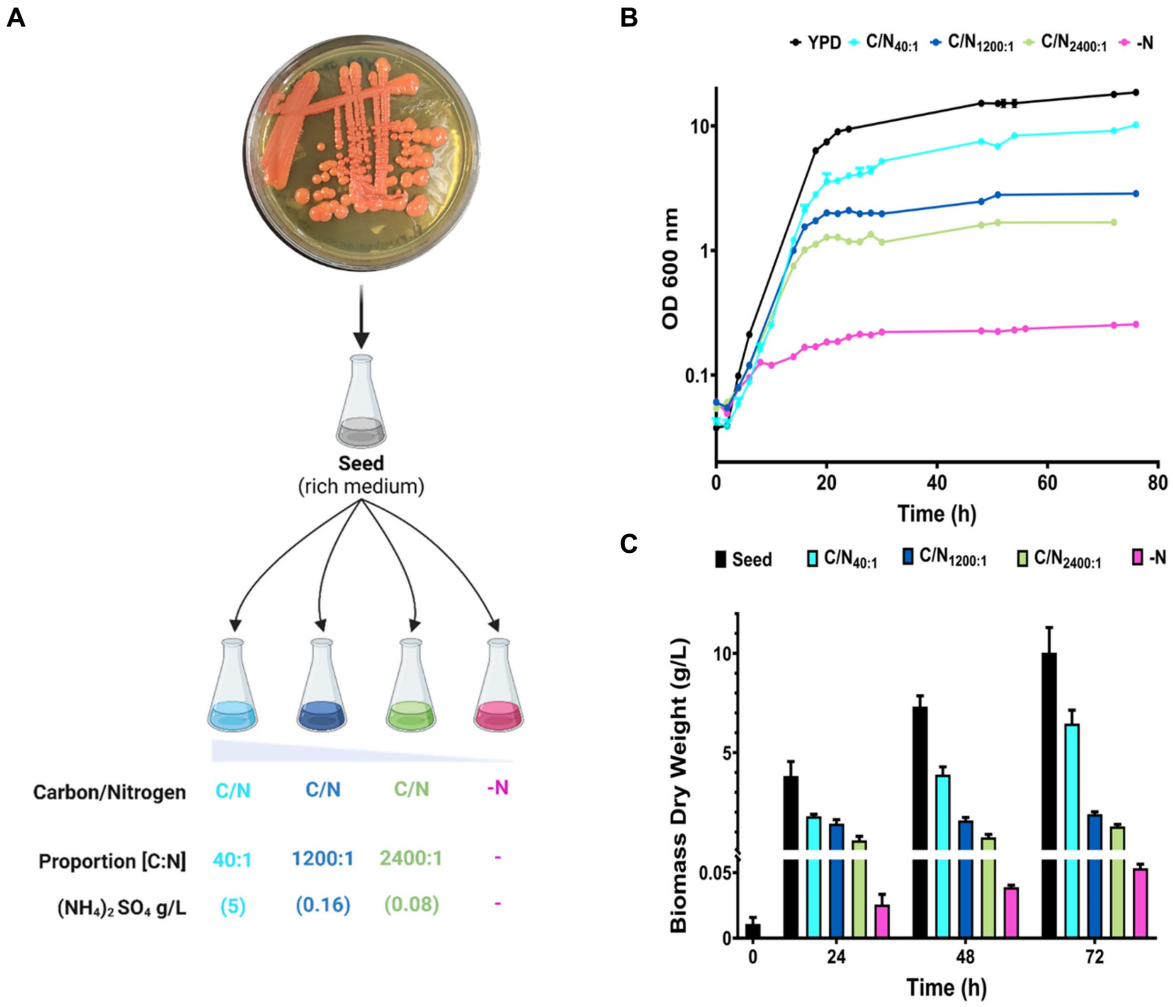
Growth curves and biomass production of *R. mucilaginosa* M94C9 varying the carbon/nitrogen ratio. **(A)** Schematic representation of growth conditions with different nitrogen concentrations. The colonies of refrigerated YPD plates were used to inoculate rich medium overnight (seed). Then, the seed culture was inoculated into different proportions of C/N (glucose/ammonium) in minimal medium as follows: 40:1 (5 g/L), 1,200:1 (0.16 g/L), 2,400:1 (0.08 g/L), and -N (without ammonium). YPD was used as a control for non-lipogenic conditions. **(B)** Cells were harvested on YPD, MM C/N_40:1_, MM C/N_1200:1_, MM C/N_2400:1_, or MM -N. Growth curves were followed for more than 70 h. (C) Under these conditions, cells were collected at 0, 24, 48, and 72 h to calculate biomass dry weight. *N* = 6, and data are the mean ± standard deviation (SD).

Cultures were prepared in rich medium (YPD), and minimal medium (MM) with four different proportions of carbon/nitrogen (C/N) availability, increasing ratios by decreasing the concentration of the nitrogen source (ammonium) and maintaining an initial excess of carbon source (glucose) (Figure 2A). Decreasing nitrogen availability reduced the OD in the early stationary phase (16–20 h), being evident during the late stationary phase (48–72 h) (Figure 2B). Under nitrogen starvation (-N), cells initially exhibit growth for 6 h before the cellular arresting. Growth in each condition was confirmed by quantifying biomass production at different time intervals (Figure 2C). The conditions YPD and C/N_40:1_ produces the highest amount of biomass compared to the other conditions, pointing out that the initial nitrogen concentration limits the growth and biomass production of *R. mucilaginosa* M94C9.

Our results show that *R. mucilaginosa* M94C9 cells have an initial continuous growth in glucose with different proportions of nitrogen during the exponential growth phase. The cells enter a quiescent state, defining the early stationary phase between 16–20 h (YPD, C/N_40:1_, C/N_1200:1_, and C/N_2400_), depending on the initial nitrogen concentration, while the stationary phase is evident at 48 h. This phenomenon is also reflected in the accumulated biomass, which depends on the amount of initial available nitrogen. Therefore, nitrogen deficiency influences the early stationary phase in *R. mucilaginosa* as a critical factor. Nitrogen depletion triggers physiological adaptations such as autophagy and nitrogen recycling, and reorients carbon flux towards store compounds, as reported in various yeast species (Tesnière et al., 2015). Thus, nitrogen depletion affects the biomass production of *R. mucilaginosa* M94C9, which should trigger physiological changes, providing an adaptive strategy to conditions of nitrogen scarcity.

### 3.2 Nitrogen limitation triggers changes in the ultrastructures of *R. mucilaginosa* M94C9

**Figure 3:**
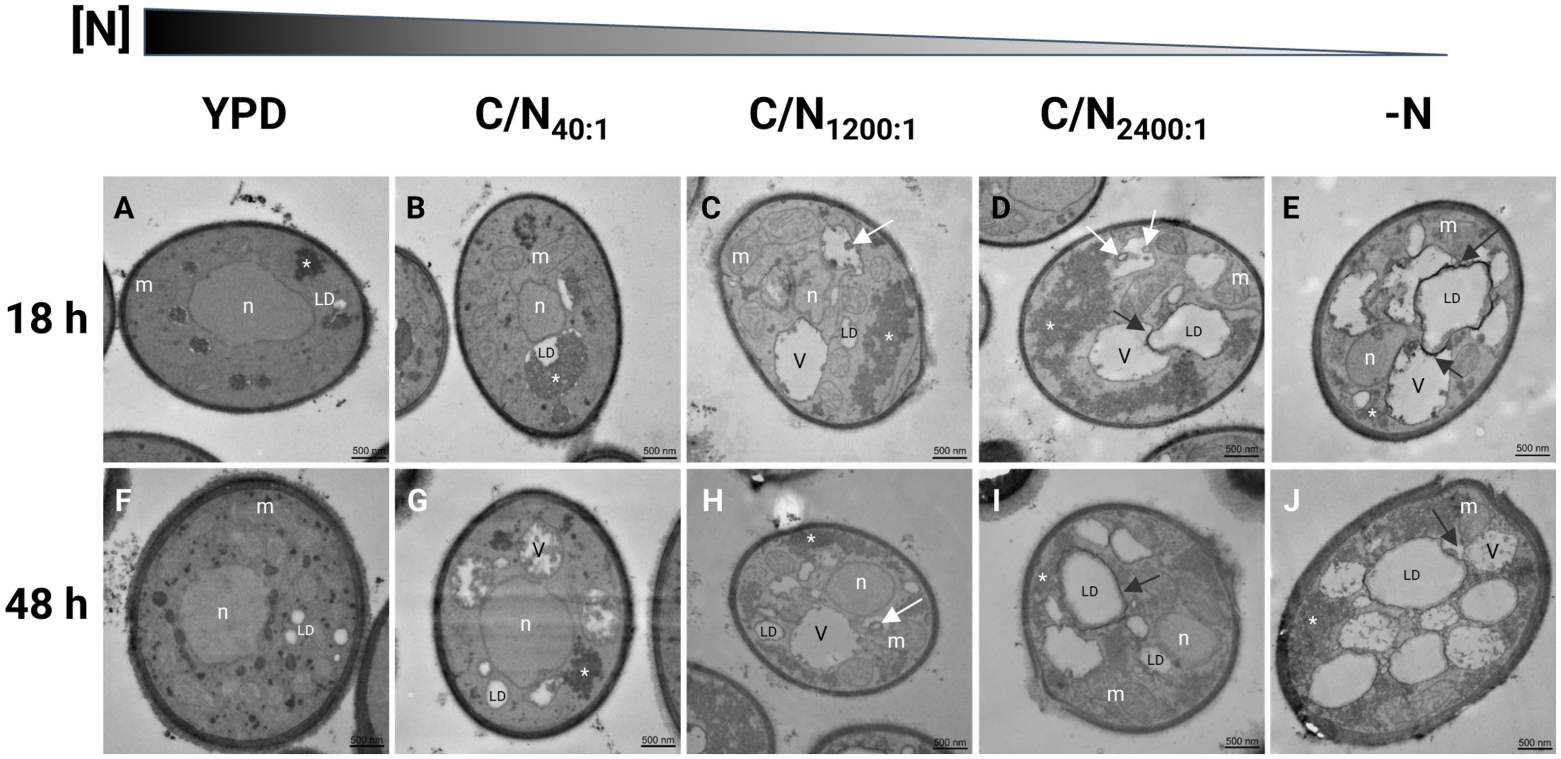
Transmission electron microscopy in *R. mucilaginosa* M94C9 cells cultivated in different carbon/nitrogen ratios reveals changes in the organization of cellular ultrastructures. **(A–E)** Cells were grown to early stationary phase (18 h) or **(F–J)** late stationary phase (48 h) in rich medium (YPD), minimal medium with C/N_40:1_, C/N_1200:1_, C/N_2400:1_, or -N. Ultrastructural features in yeast cells as previously reported by Weisman (2003), Shpilka et al. (2015), Ren et al. (2020), Schäfer et al. (2020). White arrows: vesicles. Black arrows indicate autophagy of lipid droplets (LD) or lipophagy. V, vacuole; n, nuclei; m, mitochondria; *, electron-dense granular zones. Scale bars represent 500 nm.

Nitrogen can be limited by the composition of the growth medium. Some adaptations to nitrogen limitation can be seen as ultrastructural changes that include the emergence of lipid droplets (LDs) (Shpilka et al., 2015; Hariri et al., 2017). These studies were conducted in *S. cerevisiae*, and we hypothesize that similar structural changes could occur in *R. mucilaginosa*. Therefore, the cell ultrastructures of strain M94C9 under different conditions of nitrogen availability were analyzed by transmission electron microscopy (TEM) to observe the formation of LDs as evidence of a lipogenic effect in the conditions described at two time points of the culture, early stationary phase (18 h) and stationary phase (48 h) (Figure 3). In YPD, the volume occupied by the electron-lucent spaces of the LDs is limited in the cells (Figures 3A,F). Several mitochondria are visible in this condition, and the cytoplasm appeared more homogeneous and darker than the cells growing in nitrogen limitation conditions. In the early stationary phase (Figure 3A), cells show condensed areas of electron-dense, dark granules that often appear within electron-lucent, light areas reminiscent of LDs. In the late stationary phase (Figure 3F), cells display more LDs, as well as rounder dark granules dispersed in the cytoplasm that are no longer contained within LDs and a thickening of the cell wall with an extended layer of outer fibrils compared to cells growing in early stationary phase (Figures 3A,F). Furthermore, vacuoles are not visible, and many mitochondria remain seemingly intact in the cytoplasm (Figure 3F).

The electron-lucent areas of the cells increase in size under nitrogen-limitation or -starvation conditions (Figures 3B–E), which is evident with the enlargement of vacuoles and LDs that occupy a considerable portion of the cytoplasm (Figures 3G–J). The cells grown in C/N_40:1_ medium show dark granules in the boundaries of LDs (Figures 3B,G), which disperse into the cytoplasm, and plasma membrane invaginations appear as nitrogen decreases (Figures 3C–E,H).

**Figure 4:**
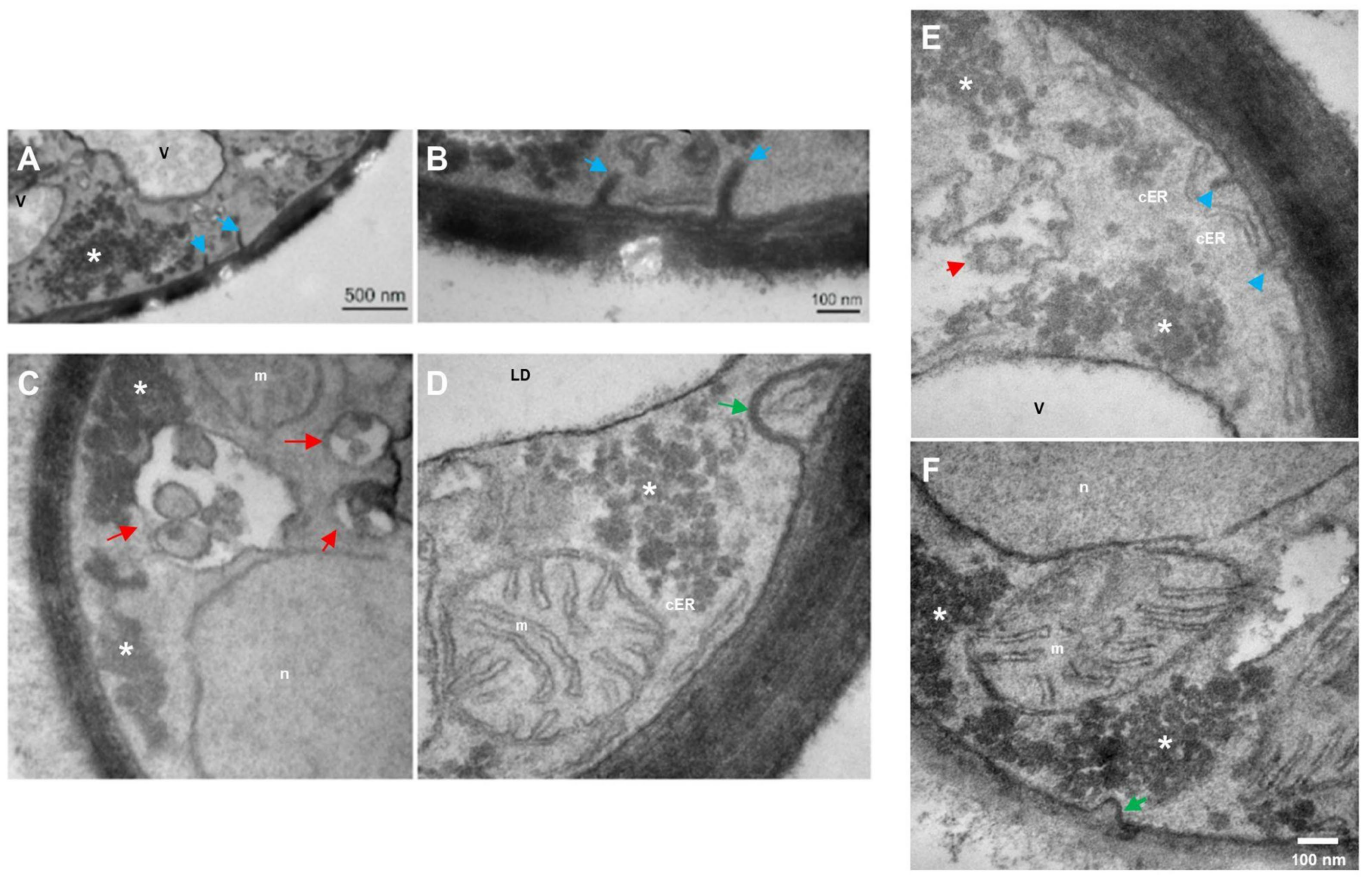
Transmission electron microscopy in *R. mucilaginosa* M94C9 cells cultivated under nitrogen starvation reveals changes in the organization of cellular ultrastructures. **(A–F)** Under -N condition at 48 h, prominent linear subcellular structures at the plasma membrane (blue arrows), endosomes (red arrows), cortical endoplasmic reticulum (cER) structure, and intermediate endocytic sites (green arrow) as previously reported ultrastructures in yeast (Strádalová et al., 2009; Buser and Drubin, 2013; Marini et al., 2020). V, vacuole; LD, lipid droplets; n, nuclei; m, mitochondria; *, electron-dense granular zones. Scale bars represent 500 or 100 nm.

Under nitrogen starvation at 48 h (Figure 4), cells exhibit linear invaginations, resembling small projections of the plasma membrane (Figures 4A,B,E), as well as endosomes (Figures 4C,E) and intermediate endocytic sites in the plasma membrane (Figures 4D,F).

### 3.3 LDs fuse and amplify in *R. mucilaginosa* M94C9 under nitrogen-limitation conditions

LDs are conserved cellular organelles that regulate lipid metabolism and impact several cellular functions. The dynamics of the production of neutral lipids were evaluated to identify the interval that increases the accumulation of LDs in *R. mucilaginosa* M94C9 under contrasting conditions of nitrogen availability (seed, C/N40:1, and -N). Lipid neutral accumulation was detected based on the intensity of the relative fluorescence (lipid index) of the compound Bodipy 493/510^Ⓡ^, using a spectrofluorometer as described in Materials and Methods. The data indicate that *R. mucilaginosa* M94C9 reaches the maximum lipid index during the stationary phase (48–72 h) under the C/N_40:1_ or -N condition (Table 3).

**Table 3:** Dynamics of lipid droplet accumulation in *R. mucilaginosa* M94C9 grown in C/N_40:1_ or -N during different time intervals.

Time	0 h	24 h		48 h		72 h	
Medium	Seed	C/N_40:1_	-N	C/N_40:1_	-N	C/N_40:1_	-N
Lipid index	73.9	205.2	246.7	184.8	300.9	210.3	286.9

**Figure 5:**
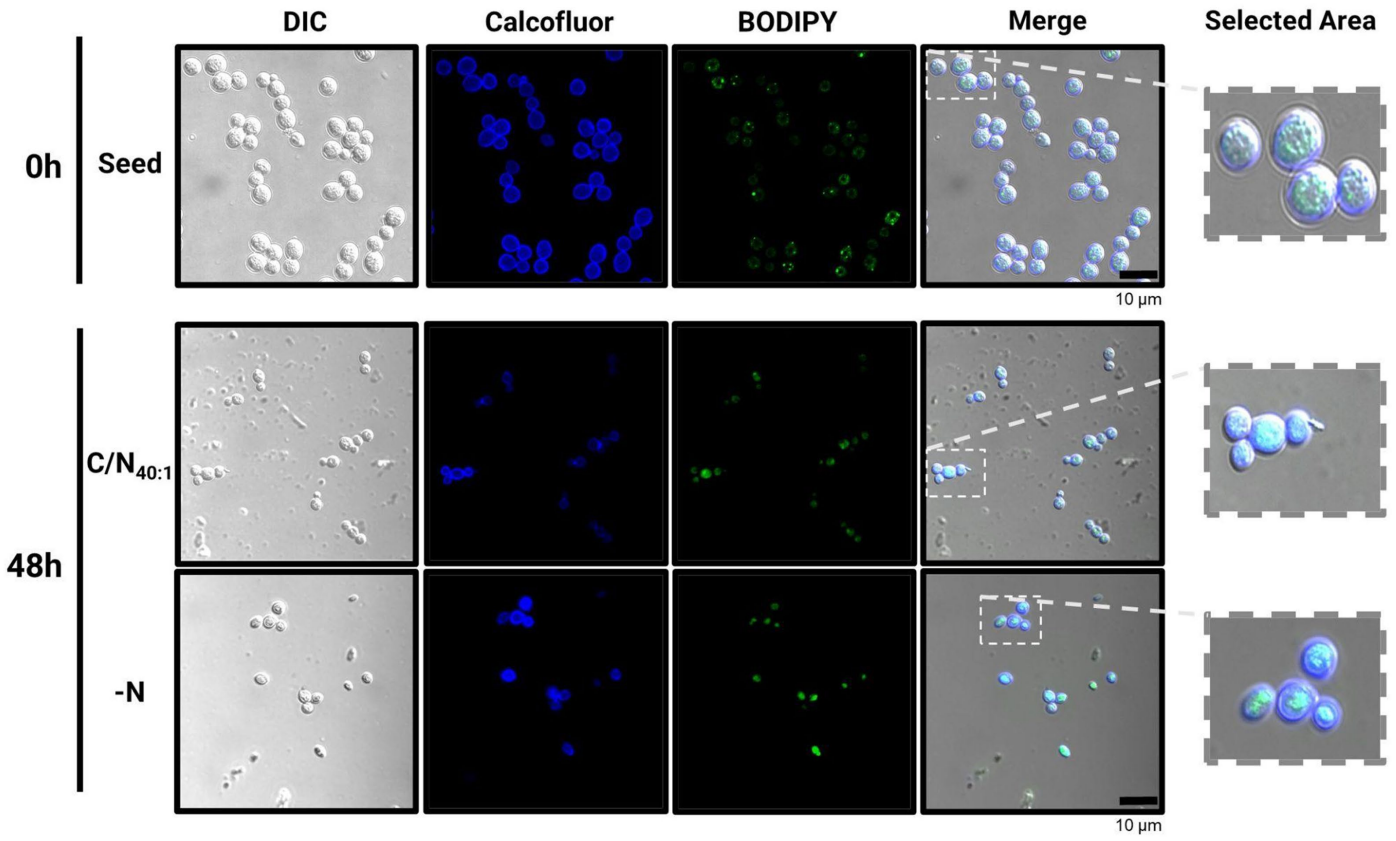
Confocal microscopy analysis in *R. mucilaginosa* M94C9 reveals fusion and amplification of LDs under C/N_40:1_ or -N condition. Cells harvested for neutral lipid staining under seed, C/N_40:1_ or -N condition. From left to right: Differential Interference Contrast (DIC), Calcofluor, Bodipy, and merging of the Calcofluor and Bodipy signals. Scale bars represent 10 μm.

These results were validated by preparing cell samples with Bodipy and observing the accumulation of intracellular LDs by confocal microscopy (Figure 5). Small LDs were observed under the seed condition (0 h), which fuse and amplify under the C/N_40:1_ or -N condition, evidencing that both conditions induce lipogenesis after 48 h. However, under the C/N_40:1_ condition, a large amount of biomass accumulates compared to the -N condition (Figure 2C), which can be advantageous in producing neutral lipids on a large scale.

### 3.4 Identification of selected genes involved in the fatty acid and triacylglycerol synthesis pathway and their expression profiles in *R. mucilaginosa* M94C9 under lipogenic conditions

The sequences of genes and proteins involved in *de novo* lipid biosynthesis of *R. mucilaginosa* strains have yet to be completely identified. Most of the studies of *R. mucilaginosa* have focused on identifying the gene sequences of the enzymes involved in the biosynthesis of carotenoids. Therefore, an *in silico* analysis was performed to find the sequences encoding acetyl-CoA carboxylase (Acc1), the beta subunit of fatty acid synthase (Fas1), the alpha subunit of fatty acid synthase (Fas2), and diacylglycerol O-acyltransferase 1 (Dga1), which have an essential role in the fatty acid (FAs) and TAGs synthesis pathways (Figure 1). The amino acid sequences of Acc1, Fas1, Fas2, and Dga1 of the yeasts *S. cerevisiae*, *R. toruloides*, and *Y. lipolytica* were used to find the genes *RmACC1*, *RmFAS1*, *RmFAS2*, and *RmDGA1* in the genome of *R. mucilaginosa* (ATCC58901 v1.0), which is deposited in the MycoCosm platform. The amino acid sequences of Acc1, Fas1, Fas2, and Dga1 were aligned with Clustal Omega and edited using MUSCLE as described in Material and Methods. Likewise, the identity percentage of each amino acid sequence was obtained (Table 4). The amino acid sequences of Acc1, Fas1, Fas2, and Dga1 are highly conserved between *Rhodotorula* spp. (more than 84%), while the sequences of *S. cerevisiae* and *Y. lipolytica* Fas2 show weak identity with the *R. mucilaginosa* sequence (10%). A comparison of *R. mucilaginosa* Fas1 sequence shows conserved regions expected for the homologs of *R. toruloides* (90%) and *Y. lipolytica* (52%).

**Table 4:** Percentage identity between the amino acid sequences of *R. mucilaginosa* Acc1, Fas1, Fas2, and Dga1, and homologous enzymes in other yeasts.

Protein function	*S. cerevisiae*	*R. toruloides*	*Y. lipolytica*
Acetyl-CoA carboxylase 1	Acc1 (55%)	RHTO_02004 (87%)	YALI0C11407g (57%)
Fatty acid synthase subunit 1	Fas1 (11%)	RHTO_02032 (90%)	YALI0B15059g (52%)
Fatty acid synthase subunit 2	Fas2 (10%)	RHTO_02139 (86%)	YALI0B19382g (10%)
Acyl-CoA diacylglycerol O-acyltransferase	Dga1 (40%)	RHTO_01962 (84%)	YALI0E32769g (51%)

**Figure 6:**
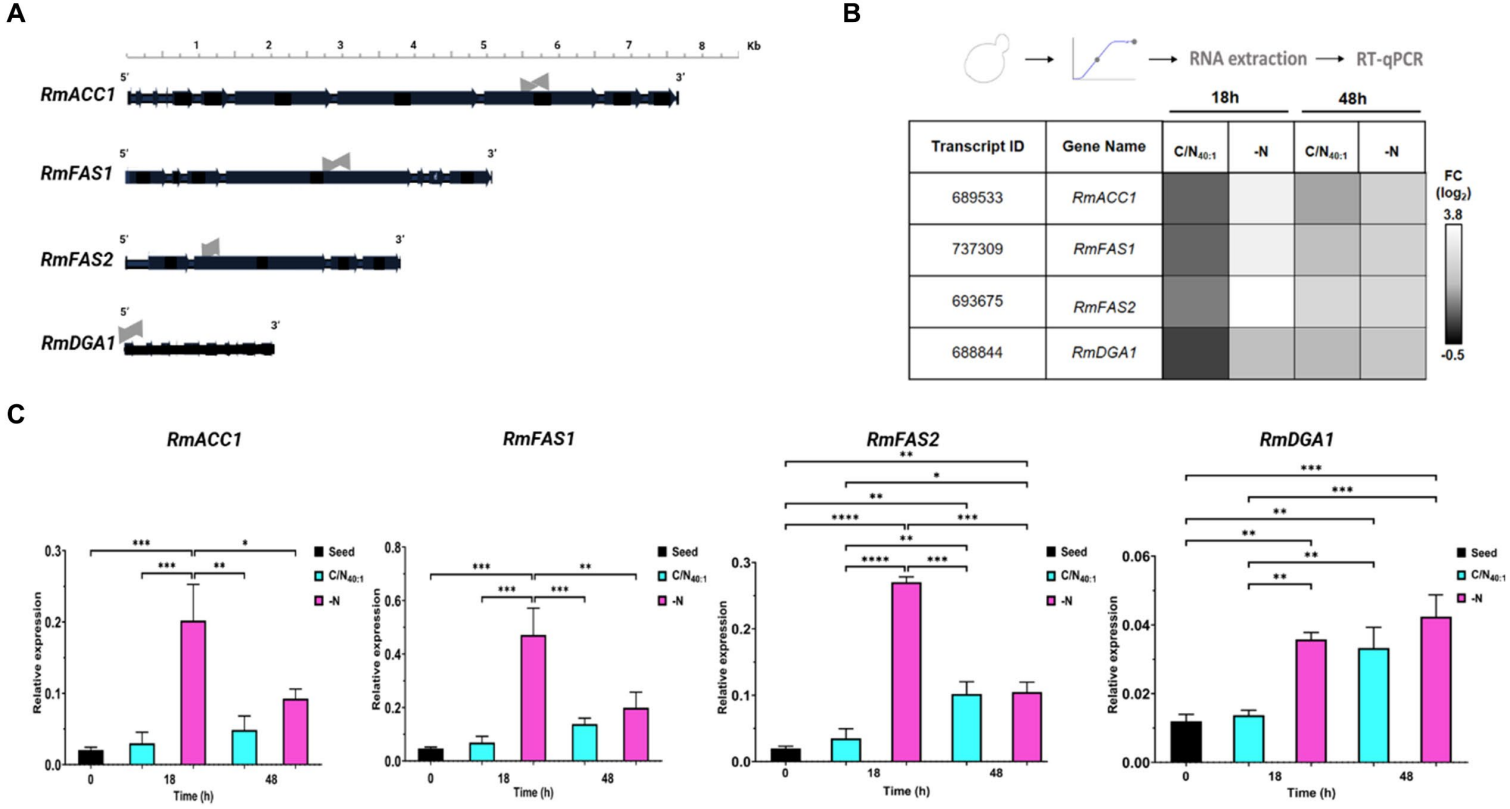
*In silico* analysis to identify open reading frames of *RmACC1*, *RmFAS1*, *RmFAS2*, and *RmDGA1* genes and their expression profiles under lipogenic conditions. **(A)** Schematic representation of genes of *RmACC1*, *RmFAS1*, *RmFAS2*, and *RmDGA1*, including exons (horizontal black arrows) and introns (horizontal black lines between exons). qPCR was performed using deoxyoligonucleotides amplifying exon regions (horizontal gray arrows). **(B)** Total RNA was extracted from cells cultivated in C/N_40:1_ or -N during the early stationary phase (18 h) and stationary phase (48 h) to be analyzed by RT-qPCR. The data obtained from the transcripts were normalized against the expression level of the actin gene (*RmACT1*). The normalized data is shown in a heatmap. The first two columns denote the ID and gene names, while the rest indicate the conditions. The fold change (log_2_) is color-coded: black for down-regulation and white for up-regulation. **(C)** The data in the bar graph shows 0 h (seed), 18 h, and 48 h of culture in C/N_40:1_ (cyan bars) or -N (pink bars). Error bars indicate the standard deviation (SD) of at least four biological replicates and two technical replicates (*N* = 8). Significant differences: *p*-value <0.05 (*), 0.01 (**), 0.001 (***), and 0.0001 (****).

The previous analysis allowed to find the genes of *RmACC1* (7,574 bp), *RmFAS1* (5,022 bp), *RmFAS2* (3,754 bp), and *RmDGA1* (2075 bp) (Figure 6A), which allowed the design of deoxyoligonucleotides to measure the expression of each messenger RNA (Table 2).

Since nitrogen is an essential nutrient, and its availability has numerous consequences for transcription levels in lipid biosynthetic pathways, the gene expression of *RmACC1*, *RmFAS1*, *RmFAS2*, or *RmDGA1* was detected by RT-qPCR analysis of samples obtained at 0 h (seed), 18 h and 48 h under C/N_40:1_ or -N condition (Figures 6B,C). The heatmap shows that the transcript of *RmACC1*, *RmFAS1*, *RmFAS2*, or *RmDGA1* is down-regulated in C/N_40:1_ at 18 h, and the expression of these genes increases in C/N_40:1_ at 48 h. In the -N condition, expression of *RmACC1*, *RmFAS1*, *RmFAS2*, and *RmDGA1* is up-regulated in both intervals, being more evident at 18 h than at 48 h (Figure 6B). Basal expression is observed when the seed (0 h) and C/N_40:1_ (18 h) conditions are compared; however, basal expression increases in C/N_40:1_ at 48 h. As expected, the expression of *RmACC1*, *RmFAS1*, *RmFAS2*, and *RmDGA1* increases significantly in the -N condition at 18 h (Figure 6C). Therefore, the genes related to fatty acid biosynthesis are up-regulated under C/N_40:1_ (48 h) or -N (18 h and 48 h), confirming that these conditions promote lipogenesis at the molecular level.

### 3.5 Fatty acids profile and enzymatic transesterification of yeast’s oil for FAEEs production

**Figure 7:**
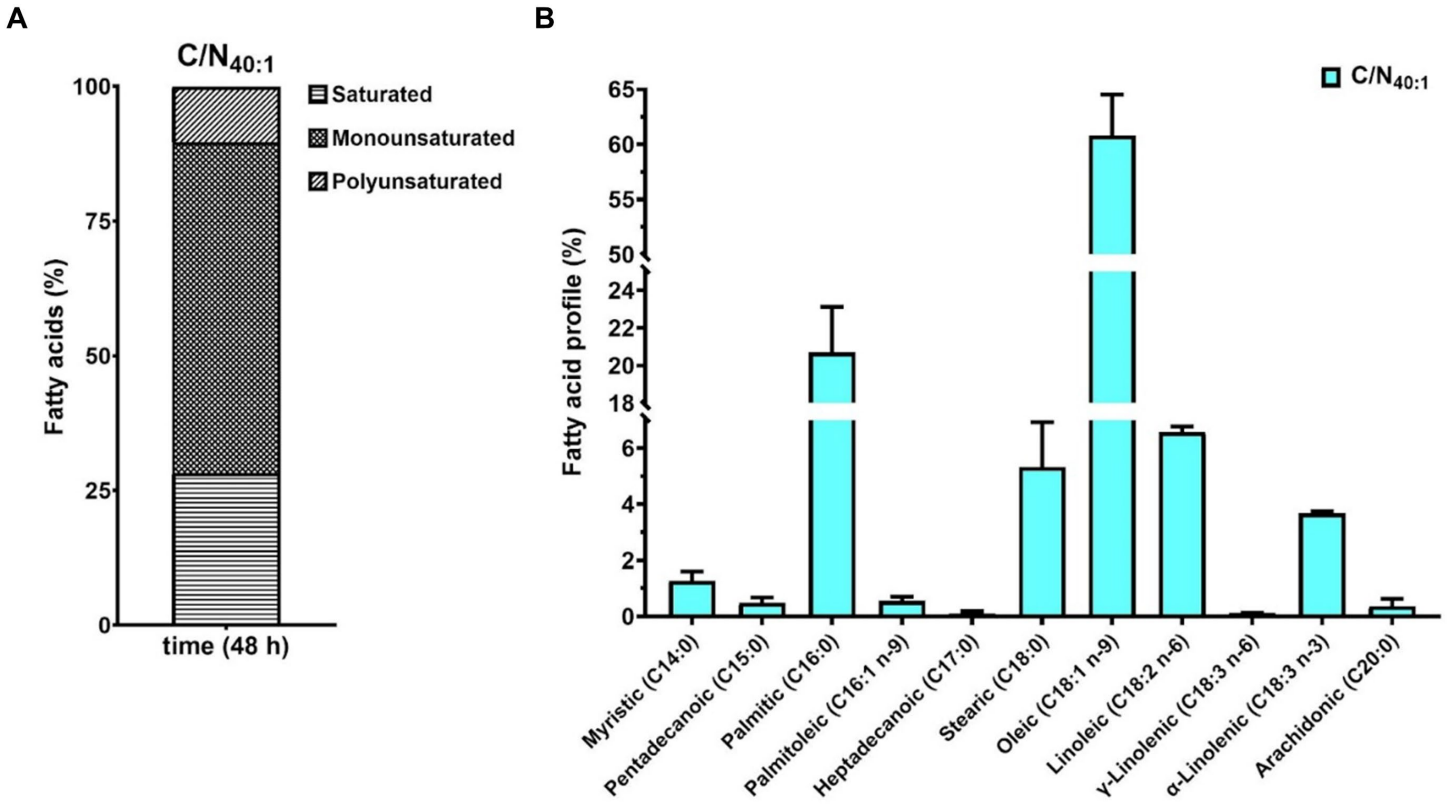
Fatty acid compositional profiles of *R. mucilaginosa* M94C9 under C/N_40:1_ during the stationary phase. **(A)** Percentage of polyunsaturated, monounsaturated, and saturated fatty acids. **(B)** Percentage of each fatty acid is indicated by C14:0, C15:0, C16:0, C16:1 n-9, C17:0, C18:0, C18:1 n-9, C18:2 n-6, γ-C18:3 n-6, α-C18:3 n-3, and C20:0. All data are presented as a mean ± SD of three biological replicates.

Oleaginous yeasts produce LDs that store intracellular neutral lipids, such as TAGs, which are composed of saturated, monounsaturated, and polyunsaturated fatty acids (SFAs, MUFAs, and PUFAs). To evaluate the fatty acids (FAs) present in the TAGs produced by *R. mucilaginosa* M94C9, the free FAs (FFAs) profile was determined in cells collected under C/N_40:1_ at 48 h, which is a lipogenic condition with biomass production through gas chromatography–mass spectrometry (GC–MS) as described in Materials and Methods. The results showed that MUFAs (61.4%) are the most prevalent, followed by SFAs (28.3%) and PUFAs (10.3%) (Figure 7A). Oleic (18:1) and palmitic (16:0) acids were the dominant FFAs, representing 60.8 and 20.7% of total FFAs, respectively (Figure 7B).

**Figure 8:**
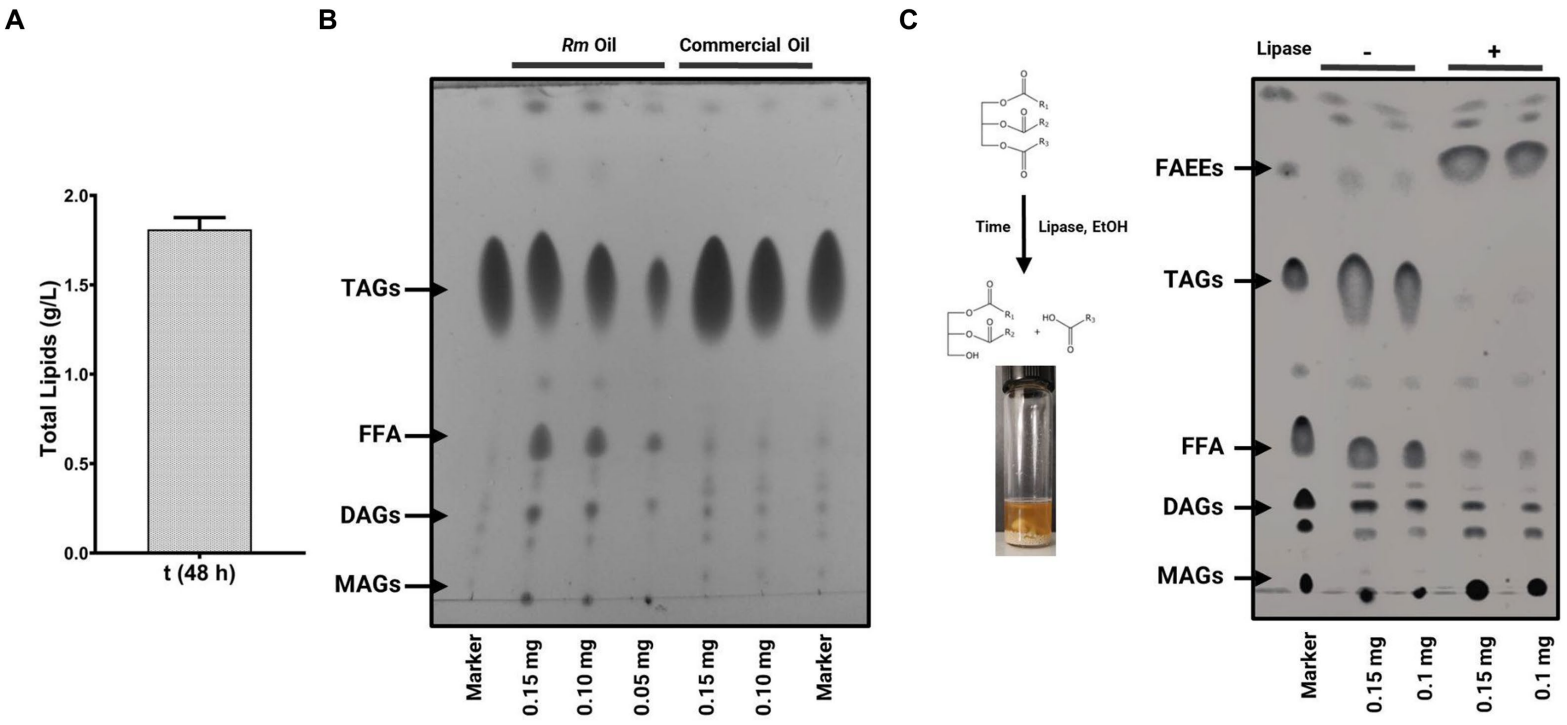
Lipid production and oil quality of *R. mucilaginosa* M94C9 as feedstock to produce biodiesel. **(A)** Total lipid extracts quantity of cells grown under the lipogenic condition (48 h). **(B)** Thin-layer chromatography (TLC) analysis of *R. mucilaginosa* M94C9 (*Rm*) oil under the lipogenic condition in stationary phase. Lane 1, marker with monoacylglycerols (MAGs), diacylglycerols (DAGs), free fatty acids (FFA), triacylglycerols (TAGs); lane 2–4, lipid extracts of *Rm* in different concentrations (0.15, 0.10 or 0.05 mg of the diluted oil); lane 5–6, commercial oil; lane 7, marker. **(C)** TLC analysis of *Rm* oil alcoholysis (ethanol) and transesterification reactions performed with Novozym 435 lipase after two days. Lane 1, marker with MAGs, DAGs, FFA, TAGs, and fatty acid ethyl esters (FAEEs); lane 2–3, lipid extracts without lipase (−); lane 4–5, lipid extracts with lipase (+).

On the other hand, it is known that the chemical properties of the TAGs that OY synthesizes are similar to the fatty acids obtained from vegetable oils used to elaborate fatty acid ethyl or methyl esters (FAEEs or FAMEs, respectively). Therefore, the TAGs of OY are a renewable and alternative source to produce biodiesel. To explore the biotechnological potential of *R. mucilaginosa* M94C9 as feedstock to produce TAGs, total lipids, and oil quality were evaluated (Figure 8), as described in Materials and Methods. A total lipid content of 1.81 g/L was obtained under lipogenic conditions (48 h), representing 46.5% of the dry cell weight (Figure 8A). The thin-layer chromatography (TLC) of Figure 8B shows the separation of yeast oil vs commercial oil. The TLC reveals that *R. mucilaginosa* M94C9 oil has a wide variety of lipids, including MAGs, DAGs, FFA, and TAGs. The major components of yeast M94C9 oil are TAGs, followed by FFA and DAGs (Figure 8B). Then, the conversion of TAGs to FAEEs was analyzed through a transesterification reaction using an immobilized lipase 435 (Novozymes) as a catalyst (Figure 8C). After 48 h of lipase reaction, 93% of the TAGs were converted to FAEEs.

## 4 Discussion

Antarctic yeasts have biotechnological interest due to their production of neutral lipids (Rossi et al., 2009; Amaretti et al., 2010; Viñarta et al., 2016). Antarctic *Rhodotorula* spp. are excellent producers of lipids with commercial value (Villarreal et al., 2018; Maza et al., 2020; Viñarta et al., 2020). Recently, *R. mucilaginosa* has attracted attention due to its promising lipid industrial applications, which could be used to replace high-value oils and as feedstocks for the production of biodiesel in the future (Béligon et al., 2016; Shields-Menard et al., 2018; Ghazani and Marangoni, 2022; Li et al., 2022; Tsai et al., 2022). However, our understanding of the physiological stimuli triggering lipid accumulation in yeasts isolated from extreme environments remains limited (Buedenbender et al., 2020; Segal-Kischinevzky et al., 2022). This work constitutes the first characterization of the Antarctic yeast *R. mucilaginosa* M94C9 under different nitrogen availability conditions with a focus on biomass production, lipid droplet dynamics, intracellular ultrastructure, expression profile of selected genes involved in lipogenesis, fatty acids biosynthesis, and biotechnological potential as feedstock for oleochemical production.

### 4.1 The intracellular ultrastructural rearrangements as adaptive responses to low nitrogen availability in *R. mucilaginosa* M94C9

**Figure 9:**
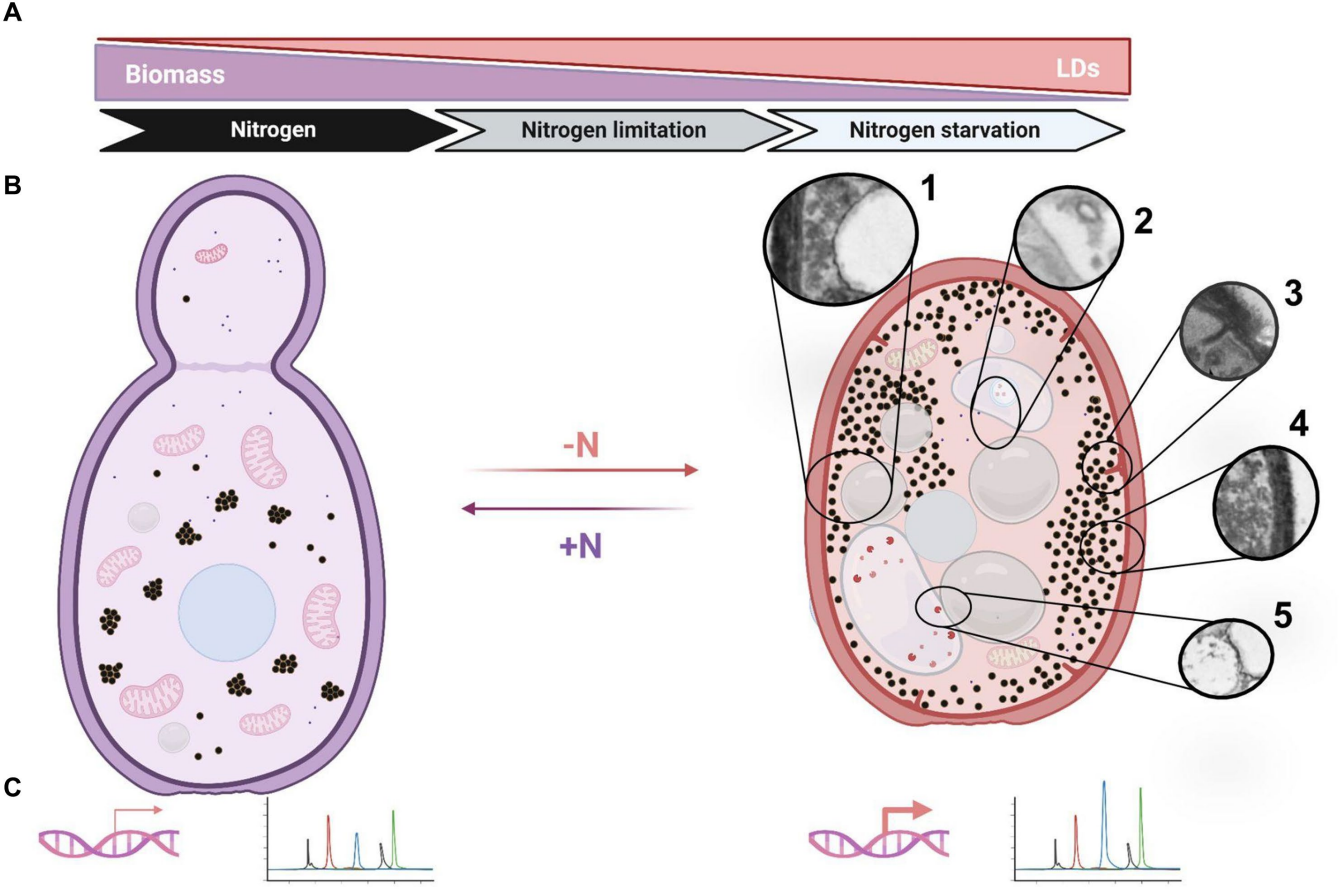
Schematic representation of cellular responses in *R. mucilaginosa* M94C6 under nitrogen stress. **(A)** The diagram depicts the induction of lipogenesis by nitrogen availability (nitrogen limitation or starvation), which increases lipid droplets (LDs) formation. **(B)** Yeast transitioning from a nitrogen-rich environment (left panel) to a nitrogen-scarce environment (right panel). Nitrogen-depleted (-N) yeast exhibit cytoplasmic compaction, as evidenced by the reorganization of cellular structures (left vs. right panel). The cytoplasmic rearrangements include the formation of large LDs (1), vacuoles containing vesicles and endosomes (2), eisosomes (3), granules dispersing to the cytoplasmic periphery (4), and docking/fusion events between LDs and vacuoles or other organelles (5). **(C)** The induction of lipogenesis is characterized by the up-regulation of key genes, such as *RmACC1*, *RmFAS1*, *RmFAS2*, and *RmDGA1*, which enhance the production of oleic and palmitic acids. All these findings represent an adaptive response to low nitrogen stress, potentially implementing as a protective mechanism to ensure cell viability (quiescence/G0) under nitrogen starvation and restoring the cell cycle (re-enter G1) when favorable environmental conditions with available nitrogen sources emerge (+N). Created using BioRender.com, accessed on 26 March 2024.

The lipid accumulation in OY is promoted by metabolism imbalance resulting from a deficiency of specific nutrients in the growth medium (Robles-Iglesias et al., 2023). Among these nutrients, phosphate and nitrogen may be included (Kolouchová et al., 2016). Nitrogen limitation conditions represent a clear nutritional stress that impacts growth and biomass accumulation, triggering lipogenesis in several OY, including *Rhodotorula* spp. (Braunwald et al., 2013; Han et al., 2016; Segal-Kischinevzky et al., 2022). Particularly, it has been remarked that high C/N ratios enhance lipid accumulation (Poontawee et al., 2023). The observations in *R. mucilaginosa* M94C9 indicate notable changes in the LDs, coupled with discernible alterations in other cellular ultrastructures (Figures 9A,B).

This phenomenon correlated with a decrease in nitrogen availability. After 18 h in rich medium (YPD), TEM unveiled metabolically active cells characterized by abundant mitochondria, a distinct nucleus, and an accumulation of electron-dense granular structures concentrated in distinct cytoplasmic regions. These electron-dense structures, which resemble glycogen grains (Wilson et al., 2010) scatter into more dispersed areas of the cytosol under nitrogen limitation or starvation conditions in *R. mucilaginosa* M94C9, which could play an essential role during nitrogen stress response. In *S. cerevisiae*, glycogen synthesis is stimulated by the low availability of nutrients, including carbon, nitrogen, phosphorus, and sulfur. The accumulation of glycogen serves as a mechanism for long-term energy storage during starvation conditions, ensuring survival until the onset of the sporulation process (Lillie and Pringle, 1980; Wilson et al., 2010). On the other hand, larger LDs were observed in nitrogen starvation compared to the different conditions, increasing their abundance within the stationary phase of growth at 48 h, which occasionally has electron-dense content and presents a distinctive lipid monolayer (Figure 9B). Conversely, LDs appear smaller and fragmented in the stationary phase of cells cultivated under the non-lipogenic condition (YPD) compared to cells grown in the lipogenic conditions (nitrogen-limitation or -starvation). After 48 h of growth in YPD, glucose is almost depleted in the rich medium. This depletion causes the cells to switch to the catabolism of LDs via β-oxidation under glucose-depleted conditions, providing the necessary energy for cellular maintenance (Kurat et al., 2006; Marini et al., 2020).

It is known that macroautophagy is prevalent in a glucose-free medium, characterized by the formation of double-membraned autophagosomes in the cytoplasm that fuse with vacuoles. However, microphagy is predominant under our experimental conditions involving high glucose as a starting point and varying nitrogen availability or nitrogen starvation. This process involves the direct internalization of LDs or ER into vacuoles, bypassing the formation of autophagic bodies (Schuck et al., 2014; van Zutphen et al., 2014; Shpilka et al., 2015; Ren et al., 2020; Schäfer et al., 2020). The vacuoles are dynamic organelles with morphological changes in response to different stresses. Vacuoles fuse during the stationary phase in response to nutrient starvation, triggering autophagy (Takeshige et al., 1992; Meaden et al., 1999; Weisman, 2003; Li and Kane, 2009). Probably, macroautophagy and microphagy may occur simultaneously under nitrogen starvation conditions (-N) in cells grown at 48 h, as evidenced by the observation of membrane elongation that incorporate LDs and organelles such as vacuoles and the formation of small circular vesicles within translucent structures (Figure 9B). However, as previously described, these interactions between membrane elongation, LDs, vacuoles, and nucleus could also be contact sites for LDs biogenesis (Hariri et al., 2017).

Another notable cellular phenomenon observed during nitrogen starvation at 48 h is the emergence of plasma membrane linear invaginations, previously discovered and termed “eisosomes,” which play a pivotal role in lipid and plasma membrane homeostasis, as well as in responses to various stresses such as hyperosmotic conditions, glucose depletion, or nitrogen starvation (Moor and Mühlethaler, 1963; Dupont et al., 2010; Gournas et al., 2018; Moharir et al., 2018; Babst, 2019; Zahumensky and Malinsky, 2019; Marini et al., 2020). This phenomenon begins with a dome-shaped indentation of the plasma membrane, which then further invaginates, coinciding with long furrows in the plasma membrane in the stationary phase that later can be smaller primary endosomes (Strádalová et al., 2009; Buser and Drubin, 2013). Hence, nitrogen stress induces ultrastructural alterations, including the enlargement of LDs and vacuoles, dispersion of granules, and the formation of eisosomes, vesicles, and endosomes (Figure 9B). These changes likely represent cellular processes crucial for the adaptation of stress-tolerant yeasts, contributing to their survival under adverse conditions.

### 4.2 Nitrogen depletion induces changes in LDs size and reduces the cell volume of *R. mucilaginosa* M94C6

High C/N ratios induce a shift in metabolic flux, favoring TAGs production deposited within LDs, which are conserved cellular organelles that regulate lipid metabolism and impact several cellular functions (Walther and Farese, 2012). Our findings in *R. mucilaginosa* M94C9 suggest an increase in the size of LDs depending on nitrogen availability. To correlate the increase in neutral lipids embedded in the LDs, we detected the relative fluorescence of Bodipy emitted by the cells cultured in the lipogenic media (C/N_40:1_ and -N) during different time intervals. Lipid index and confocal microscopy assays confirmed that *R. mucilaginosa* M94C6 increases the production of neutral lipids under nitrogen limitation and starvation conditions. We found that LDs increase in size in lipogenic conditions compared to the control condition (seed) (Figure 9B), confirming that during the stationary phase, an increase in the size of LDs in the OY can be observed (Patel et al., 2017). However, the increase in the size of LDs in *R. mucilaginosa* M94C6 depends on the C/N ratio or the initial nitrogen limitation since the increase in the size of LDs is not observed in the stationary phase (48 h) in a rich medium, as shown by TEM microscopy.

On the other hand, owing to the uncertain supply of nutrients in nature, the yeast cell-division rate must be coordinated with widely variable cell growth rates. Nitrogen limitation or starvation reduces cell size at division, delaying mitosis (MacNeill, 1994), as revealed by confocal microscopy analysis of stationary phase grown cells (C/N_40:1_) or nitrogen depletion (-N). Hence, nitrogen depletion increases LDs size and a reduction in cell volume in *R. mucilaginosa* M94C6 during the stationary phase (C/N_40:1_) or under prolonged nitrogen starvation. This response may serve as a mechanism to safeguard cell viability and facilitate future sporulation.

### 4.3 The expression profile *RmDGA1* gene as a marker of lipogenesis in *R. mucilaginosa* M94C6 under nitrogen deficient conditions

Decreased mitochondrial AMP levels affect the activation of the isocitrate dehydrogenase and trigger a series of events that lead to the production of cytosolic acetyl-CoA (Figure 1), which is critical for *de novo* lipid synthesis through different enzymes, including Acc1 (acetyl-CoA carboxylase), Fas1/2 (fatty acid synthase 1 and 2), and Dga1 (diacylglycerol acyltransferase) under nitrogen-limiting conditions in OY (Papanikolaou and Aggelis, 2011). Here, we identify the large gene-coding regions of the *RmACC1* (7.57 kb), *RmFAS1* (5.02 kb), *RmFAS2* (3.75 kb), and *RmDGA1* (2.07 kb) in *R. mucilaginosa*, which included exons and multiple short introns as others *Rhodotorula* sp., (Zhu et al., 2012). The transcripts were subsequently quantified in cells cultured at 0 h (seed), 18 h, and 48 h under nitrogen-limitation (C/N_40:1_) and -starvation (-N), which are contrasting conditions. *RmACC1*, *RmFAS1*, *RmFAS2*, and *RmDGA1* expression levels rise with culture time (0, 18, and 48 h) in the C/N_40:1_ condition. This observation suggests that under the C/N_40:1_ condition, nitrogen is gradually depleted, consequently triggering the up-regulation of these genes as the culture progresses toward the stationary phase. We assume that the increased transcription of *RmACC1*, *RmFAS1*, *RmFAS2*, and *RmDGA1* at both 18 and 48 h maintain a sustained flux of lipid biosynthesis intermediates, resulting in a gradual TAGs accumulation within LDs with significative biomass production (Figure 9C). This is supported by biomass, lipid index, and microscopy results under the C/N_40:1_ condition compared to the seed cultures. On the other hand, the expression of *RmACC1*, *RmFAS1*, *RmFAS2*, and *RmDGA1* (0 h vs 18 h) increases in the -N condition at 18 h, which subsequently decreases at 48 h. Nevertheless, the transcription of *RmDGA1* does not decrease; it remains elevated at 48 h under nitrogen starvation.

Currently, it is understood that Acc1 activity is essential for converting acetyl-CoA to malonyl-CoA during lipogenesis and/or autophagy, processes necessary for lipid production to form membranes during vacuolar fusion in the stationary phase. Conversely, it is known that the Acc1 activity diminishes in seed cultures or during the logarithmic growth phase, especially in environments abundant in nitrogen. In these conditions, the primary requirement for acetyl-CoA is directed toward histone acetylation, gene activation, and growth processes (Shi and Tu, 2015). In fact, the overexpression of *ACC1* encourages lipid accumulation in OY, such as *Y. lipolytica* (Zeng et al., 2018; Ghogare et al., 2020; Chaturvedi et al., 2021, 2022). However, repression of *ACC1* expression results in reduced cell growth and proliferation, coinciding with the depletion of intracellular lipid reserves in non-oleaginous yeasts such as *S. cerevisiae* (Gross et al., 2019). On the other hand, Fas1/2 constitutes an essential enzymatic complex responsible for fatty acid synthesis, catalyzing the conversion of acetyl-CoA and malonyl-CoA into long-chain acyl-CoA using NADPH as a reducing agent (Cui et al., 2016). Under potassium acetate starvation, the Fas1/2 complex is transported to the vacuole (Egner et al., 1993) and, during nitrogen deprivation, is degraded to maintain cell viability (Shpilka et al., 2015). Our results demonstrate that the expression patterns of *RmACC1*, *RmFAS1*, and *RmFAS2* under nitrogen-limitation and -starvation conditions are consistent with previous reports (Sailwal et al., 2023). Specifically, these genes exhibit basal expression levels during the nitrogen-rich seed cultures, thereby facilitating cell proliferation. However, the transcript of *RmACC1*, *RmFAS1*, and *RmFAS2* is induced over time, correlating with nitrogen depletion that triggers LDs accumulation, as shown in microscopy analysis under the -N condition at 18 h. Subsequently, there is a decrease in the expression of *RmACC1*, *RmFAS1*, and *RmFAS2* at 48 h, suggesting down-regulation to potentially sustain cell viability during prolonged nitrogen starvation.

The final step in the *de novo* lipid synthesis pathway entails the production of TAGs (Papanikolaou and Aggelis, 2011). The TAGs are synthesized by the enzymes Lro1 and/or Dga1, localized in the endoplasmic reticulum membrane or in LDs as Dga1 (Choudhary and Schneiter, 2020). A notable discrepancy between these enzymes lies in their activity during distinct growth phases; Lro1’s activity peaks during the exponential growth phase, while Dga1 significantly contributes to TAGs synthesis during the stationary phase (Wang, 2015). It is evident that both enzymes partake in TAGs synthesis. Nonetheless, in response to stress induced by nutrient deprivation, such as nitrogen scarcity, Dga1 appears to assume a more prominent role, with Lro1 primarily implicated in membrane phospholipid remodeling (Oelkers et al., 2002; Sandager et al., 2002; Kamisaka et al., 2013). Furthermore, in yeast, deletion of the *DGA1* gene results in the formation of aberrant LDs (Wang, 2015). Moreover, it has been documented that excessive accumulation of DAG due to upregulated lipogenesis under nitrogen starvation is deleterious for both autophagy and endomembrane systems in *dga1*Δ*lro1*Δ cells (Li et al., 2020). Our findings indicate that *RmDGA1* expression increases at the stationary phase (48 h) in the C/N_40:1_ or under the -N condition at 18 h and 48 h. Interestingly, *RmDGA1* expression is increased and sustained throughout nitrogen starvation at both time intervals, which could be linked to the relevance of Dga1 during lipogenesis and autophagy (Athenstaedt, 2011; Li et al., 2015). Therefore, under nitrogen-starved conditions, the prolonged up-regulation of the *RmDGA1* gene is a molecular indicator of LDs accumulation (Figures 9A–C), which is probably essential for the regulation of starvation-induced autophagy in *R. mucilaginosa* M94C6.

### 4.4 The oleaginous yeast *R. mucilaginosa* M94C6 is a promising feedstock for neutral lipids production

It has been established that OY can exhibit diverse FAs profiles, strongly influenced by growth conditions such as carbon and nitrogen sources in the species involved (Aloklah et al., 2014; Klug and Daum, 2014). This variability leads to notable differences in compositions among different yeasts. Our FAs profiles obtained align with those reported in the literature (Papanikolaou and Aggelis, 2011). The FAs profile analysis was conducted under the C/N_40:1_ condition during the stationary phase (48 h); the predominant FAs in *R. mucilaginosa* M94C6 were oleic acid (C18:1) and palmitic acid (C16:0), accounting for 60.8 and 20.7% of the total, respectively. In OY, the *de novo* FAs synthesis is characterized primarily by the predominant presence of C18:1, followed by C16:0, C18:2, C18:0, and palmitoleic acid (C16:1), resulting in a higher concentration of unsaturated FA, as reported in previous studies (Papanikolaou and Aggelis, 2011), which is consistent with our results. This composition of unsaturated FAs (mainly C18:1 and C18:2) can lead to the formation of reactive oxygen species. Consequently, cells store them in the form of TAGs within LDs (Eisenberg and Büttner, 2014). On the other hand, C18:1 has been shown to confer increased cold resistance in yeast, while C16:1 and C18:1 provide high-temperature tolerance and ethanol resistance (You et al., 2003; Rodríguez-Vargas et al., 2007; Kim et al., 2011; Phong et al., 2022).

Due to the prevalence of C18:1, C16:0, and C18:0 as the primary FAs derived from OY, numerous authors have highlighted the resemblance of this profile to that of vegetable oils, particularly those employed in the production of biodiesel and oleochemicals. Biodiesel production is a potential future application for the neutral lipids generated by OY (Caporusso et al., 2021). To establish a standardized biodiesel production process from a specific OY strain, it is crucial to investigate culture parameters that ensure both effective lipid accumulation and optimal growth (Lopes et al., 2020). In this context, we evaluated a lipogenic condition at 48 h, previously documented in the literature as capable of enhancing lipid synthesis in OY (Niehus et al., 2018). Implementing this culture condition resulted in extracting 1.81 g L^−1^ of total lipids, equivalent to 46.5% of the total biomass of the *R. mucilaginosa* M94C9 strain. This percentage of accumulated lipids conclusively highlights *R. mucilaginosa* M94C9 as an OY (Salvador López et al., 2022).

In comparison with recent reports on lipid accumulation in other strains of *R. mucilaginosa*, the amount of lipid obtained in this research is comparable to that achieved in other studies (0.57, 1.5, or 1.83 g L^−1^) that also conducted their cultures in flasks (Banerjee et al., 2020; da Silva et al., 2020; Tsai et al., 2022). While *R. mucilaginosa* could be a good prospect for accumulating lipids, utilizing these strains as raw material in industrial biodiesel production is currently impractical since obtaining microbial biomass is highly expensive when cultures are prepared with glucose as carbon source (Koutinas et al., 2014). Given this challenge, alternative carbon sources should be used as glycerol, which is abundant in biodiesel production waste, and can be repurposed for lipid biosynthesis and microbial biomass production (Taccari et al., 2012). In this regard, it is crucial to consider our study for enhancing lipid production under nitrogen-limitation conditions in combination with renewable and cost-effective carbon sources such as glycerol for future investigations. These studies will focus on developing strategies to evaluate growth conditions under nutritional stress to optimize the production of TAGs and biomass, thereby enabling profitable biodiesel production.

Could the neutral lipids extracted from *R. mucilaginosa* M94C9 be utilized in the production of enzymatic biodiesel? The transesterification assay performed with Novozym 435 yielded a 93% conversion of TAG into biodiesel within 48 h. Enzymatic catalysis has emerged as an environmentally friendly alternative to traditional acid- and base-catalyzed methods, which generate polluting effluents during catalyst inactivation and biodiesel washing and recovery steps (Sandoval et al., 2017). Enzymatic catalysis can produce biodiesel without the formation of undesirable by-products. However, the primary disadvantage is the high cost of lipases (Cavalcante et al., 2021).

Biodiesel production from yeast oils using enzymatic catalysis has been previously reported. For example, *R. toruloides* achieved yields of 67 to 88% in 24 h, while *Trichosporon shinodae* achieved a 90% yield in 12 h (Zhao et al., 2012; Kumar et al., 2022). In comparison, traditional methods like basic catalysis achieved around 97% yield in 10 h with *R. toruloides* oils (Thliveros et al., 2014) and 96.6% yield in 3 h with *R. mucilaginosa* R2 oils (Gohain et al., 2020). This suggests that enzymatic methods are less efficient in reaction time and cost. Nevertheless, enzymatic methods for biodiesel production are more environmentally friendly than traditional methods due to lower generation of contaminant effluents, as they do not require catalyst deactivation or washing stages. Therefore, *Rhodotorula* oils could serve as a raw material for future pilot-scale enzymatic biodiesel production, however, it is necessary to continue standardizing the method to reduce costs and optimize yield.

### 4.5 Concluding remarks

Our study reveals that nitrogen limitation and starvation trigger significant changes in the cellular growth and lipid metabolism of the Antarctic yeast *R. mucilaginosa* M94C9, including morphological and ultrastructural alterations and differential gene expression related to lipogenesis. The major components of the oil from strain M94C9 are TAGs, followed by FFA and DAGs, with oleic acid and palmitic acid being the predominant FAs. These findings offer valuable insights into cultivation strategies that could enhance lipid production in *R. mucilaginosa*. This research significantly advances our understanding of Antarctic microorganisms, providing promising insights for developing sustainable oleochemical biotechnologies, particularly under oligotrophic conditions such as nitrogen starvation, which stimulate lipid biosynthesis. However, further research will be needed on *R. mucilaginosa*. This new research should utilize omics approaches, metabolic engineering, process optimization, and assessment of economic viability at an industrial scale in the future.

## Data availability statement

The original contributions presented in the study are included in the article/supplementary material, further inquiries can be directed to the corresponding author.

## Author contributions

MR-P: Writing – original draft, Methodology, Investigation, Formal analysis, Data curation. AZ-B: Writing – review & editing, Methodology, Investigation, Formal analysis, Data curation. NT-R: Writing – review & editing, Methodology, Investigation, Formal analysis. DV-H: Data curation, Writing – review & editing, Methodology, Investigation, Formal analysis. LR-A: Writing – review & editing, Methodology, Formal analysis, Data curation. JP: Writing – review & editing, Methodology, Formal analysis, Data curation. MH: Writing – review & editing, Methodology, Formal analysis, Data curation. GS: Writing – review & editing, Methodology, Formal analysis, Data curation. CS-K: Investigation, Writing – review & editing, Formal analysis. JG: Writing – original draft, Supervision, Project administration, Methodology, Funding acquisition, Data curation, Conceptualization, Writing – review & editing, Investigation, Formal analysis.

## Footnotes

^1^https://genome.jgi.doe.gov/programs/fungi/index.jsf

^2^https://www.ebi.ac.uk/Tools/msa/clustalo/

## Glossary

OY: oleaginous yeasts
TAGs: triacylglycerols
LDs: lipid droplets
-N: nitrogen starvation
Acc1: acetyl-CoA carboxylase 1
Fas1: fatty acid synthase subunit 1
Fas2: fatty acid synthase subunit 2
Dga1: diacylglycerol acyltransferase
ACC1: gene encoding Acc1
FAS1: gene encoding Fas1
FAS2: gene encoding Fas2
DGA1: gene encoding Dga1
DAG: diacylglycerol
ER: endoplasmic reticulum
cER: cortical endoplasmic reticulum
seed: cells grown in a rich medium overnight defined as time 0 h
C/N: carbon to nitrogen ratio
V: vacuole
n: nuclei
m: mitochondria
SFAs: saturated fatty acids
MUFAs: monounsaturated fatty acids
PUFAs: polyunsaturated fatty acids
FFAs: free fatty acids
